# Glypican-3: A Novel and Promising Target for the Treatment of Hepatocellular Carcinoma

**DOI:** 10.3389/fonc.2022.824208

**Published:** 2022-02-16

**Authors:** Xiufeng Zheng, Xun Liu, Yanna Lei, Gang Wang, Ming Liu

**Affiliations:** ^1^ Department of Abdominal Oncology, West China Hospital, Sichuan University, Chengdu, China; ^2^ National Engineering Research Center for Biomaterials, Sichuan University, Chengdu, China

**Keywords:** glypican-3 (GPC3), hepatocellular carcinoma (HCC), cancer immunotherapy, immune checkpoint blockade, chimeric antigen receptor

## Abstract

Glypican-3 (GPC3) is a membrane-associated proteoglycan that is specifically up-regulated in hepatocellular carcinoma (HCC) although rarely or not expressed in normal liver tissues, making it a perfect diagnostic and treatment target for HCC. Several GPC3-based clinical trials are ongoing and recently several innovative GPC3-targeted therapeutic methods have emerged with exciting results, including GPC3 vaccine, anti-GPC3 immunotoxin, combined therapy with immune checkpoint blockades (ICBs), and chimeric antigen receptor (CAR) T or NK cells. Here, we review the value of GPC3 in the diagnosis and prognosis of HCC, together with its signaling pathways, with a specific focus on GPC3-targeted treatments of HCC and some prospects for the future GPC3-based therapeutic strategies in HCC.

## 1 Introduction

Liver cancer is the second-most cause of cancer death throughout the world (8.2% of the total) (1), and hepatocellular carcinoma (HCC) is the most common type of liver cancer. Despite significant advances in both diagnosis and treatment, only 40% of HCC is diagnosed at an early stage, and the results of treatment are often disappointing. Surgery is still the preferred treatment. However, only 5%-10% of HCC tumors are suitable for resection, and tumor recurrence occurs in a majority (50%-70%) of patients within five years of surgery. Although liver transplantation offers an alternative, the numbers of suitable donor liver sources are extremely limited, while waiting for the donor liver, the tumor may progress, which may lead to the loss of surgical opportunity or worsen the postoperative prognosis (2). Systemic chemotherapy with oxaliplatin-based regimens has been found to increase the overall survival (OS) by 1.47 months (3). Multiple tyrosine kinase inhibitors, sorafenib (4), used as first-line treatments, while lenvatinib (5) and donafenib were found to be superior to sorafenib in extending the OS in Chinese patients with advanced HCC (6).

Immunotherapy has become a powerful strategy for treating cancer. Anti-programmed cell death protein 1 (PD-1) inhibitors of nivolumab (7) and pembrolizumab (8), anti-CTLA-4 inhibitors of tremelimumab (9) and ipilimumab (10), the preliminary results showed promising antitumor activity in HCC. At present, the general trend in tumor treatment is the use of combination therapy, Atezolizumab combined with bevacizumab was found to improve the patient prognosis with an excellent objective response rate (ORR) in advanced HCC (11), and lenvatinib combined with pembrolizumab or sintilimab combined with bevacizumab showed similar results (12, 13). Nevertheless, despite the progress of current treatments, there are still limited options for effective systemic treatment of HCC. As a result, its five-year survival rate is only a dismal 18% (14). Thus, the identification of specific molecular markers and targets would assist both early diagnosis and targeted therapy.

Glypian-3 (GPC3) is a heparan sulfate proteoglycan (HSPG). There are six glypican subtypes, namely, GPCs 1-6, with similar structures consisting of a 60-70 kDa protein connected to the cell membrane by a glycosylphosphatidylinositol (GPI) anchor, 14 conserved cysteine residues, and the last 50 residues at the carboxyl end modified by the heparan sulfate (HS) side-chain. GPC3 has been implicated in a variety of processes, including cell growth, differentiation, and migration (15, 16). The specific expression of GPC3 in tumor cells has received widespread attention. Here, we discuss the relevance of GPC3 to HCC diagnosis and prognosis, and also address the signaling pathways used by GPC3 to promote HCC development, and focus on the feasibility of targeting GPC3 for treating HCC.

## 2 Relevance of GPC3 to the Diagnosis and Prognosis of HCC

**Figure 1:**
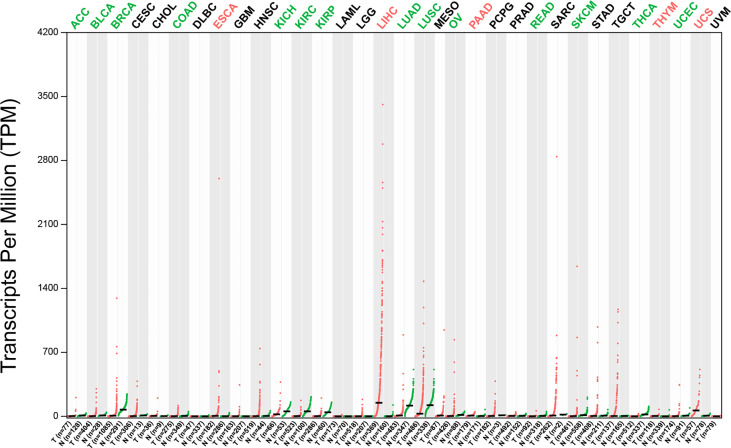
The expression profile of GPC3 across tumor samples and paired normal tissues (Dot plot). Each dot represents expression of samples. T, tumor samples; N, normal tissues; ACC, adrenocortical carcinoma; BLCA, bladder urothelial carcinoma; BRCA, breast invasive carcinoma; CESC, cervical squamous cell carcinoma & endocervical adeno; CHOL, cholangiocarcinoma; COAD, colon adenocarcinoma; DLBC, lymphoid neoplasm diffuse large B-cell lymphoma; ESCA, esophageal carcinoma, glioblastoma multiforme; HNSC, head & neck squamous cell carcinoma; KICH, kidney chromophobe cell carcinoma; KIRC, kidney renal clear cell carcinoma; KIRP, kidney renal papillary cell carcinoma; LAML, acute myeloid leukemia; LGG, brain lower grade glioma; LIHC, liver hepatocellular carcinoma; LUCD, lung adenocarcinoma; LUSC, lung squamous cell carcinoma; MESO, mesothelioma; OV, ovarian serous cystadenocarcinoma; PAAD, pancreatic adenocarcinoma; PCPG, pheochromocytoma & paraganglioma; PRAD, prostate adenocarcinoma; READ, rectum adenocarcinoma; SARC, sarcoma; SKCM, skin cutaneous melanoma; STAD, stomach adenocarcinoma; TGCT, testicular germ cell tumors; THCA, thyroid carcinoma; THYM, thymoma; UCEC, uterine corpus endometrial carcinoma; UCS, uterine carcinosarcoma; UVM, uveal melanoma.

**Figure 2:**
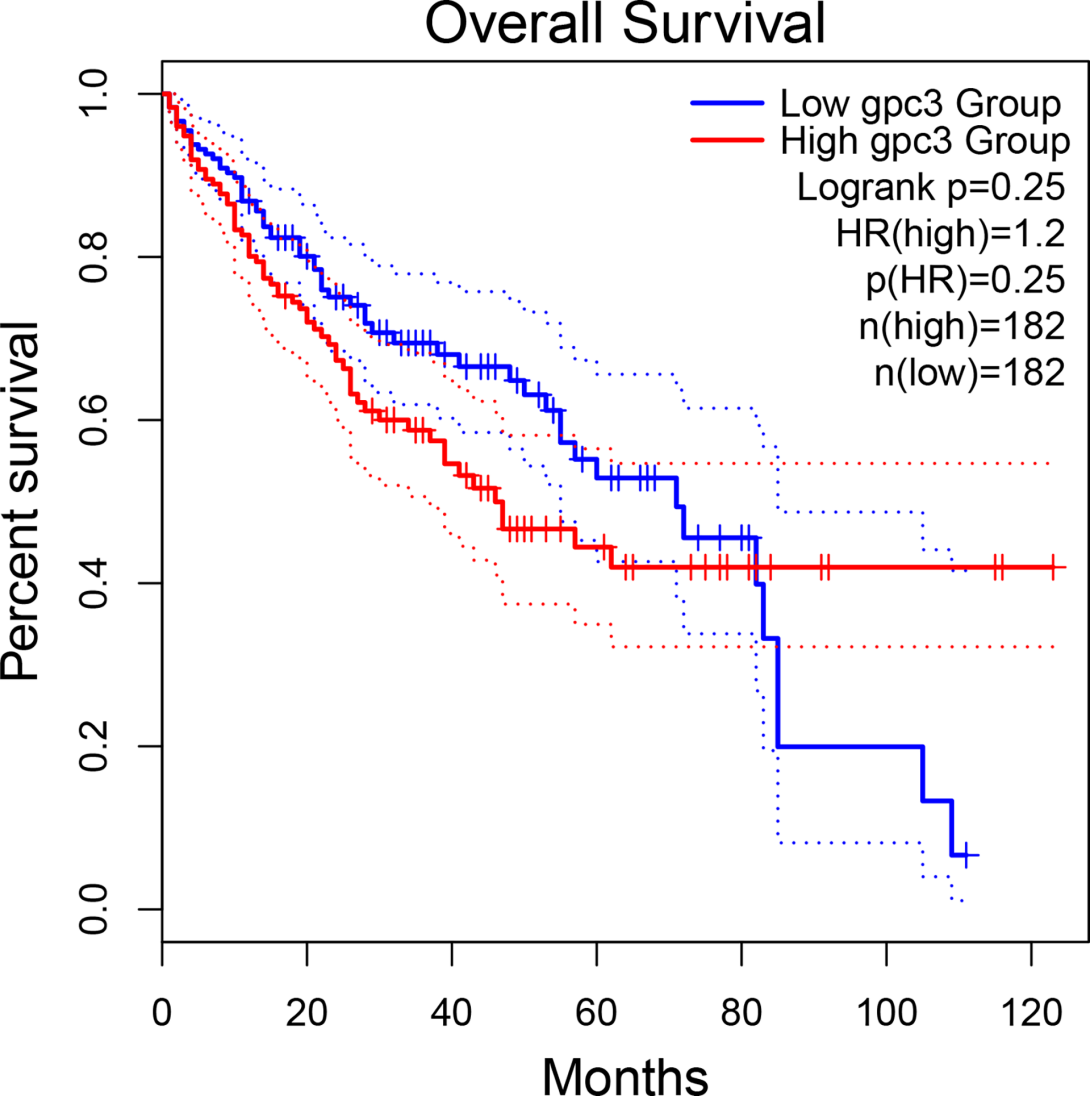
The association between GPC3 expression and HCC prognosis.

The potential of GPC3 in HCC diagnosis and prognosis is gradually being recognized. **Figure 1** compares GPC3 expression in various cancers and normal tissues (17). In1997, Hsu et al. demonstrated that MXR7 (later shown to be GPC3) was more strongly expressed in HCC than AFP (18), but was not visible in either normal liver or benign liver lesions (such as cirrhotic or dysplastic nodules) (19). Immunostaining also demonstrated the presence of GPC3 in small liver tumors (16). Currently, GPC3-targeted imaging includes positron emission tomography (PET) (20, 21), magnetic resonance imaging (MRI) (22), and near-infrared imaging (NIR) (23) for the early diagnosis of HCC, showing excellent results and high specificity in HCC. GPC3 is also found in the serum of many HCC patients but not in sera from healthy individuals or patients with hepatitis. Despite the presence of GPC3 being indicative of an HCC diagnosis, a single marker cannot meet the specificity and sensitivity requirements of clinical practice. GPC3 + HSP70 (heat shock protein 70) + GS (glutamine synthetase) is an optimal combination to distinguish early and grade 1 HCC from dysplastic nodules in cirrhosis, strengthening the diagnosis of suspected HCC, especially in a biopsy with few samples (24, 25). Other investigations have also proposed some combinations of potential markers, such as arginase-1/heppar-1/GPC3 (26), GP73/GPC3/CD34 (27), and GPC3/CD34 (28). Elevated levels of GPC3 in tumor cells is related to poor prognosis, as **Figure 2** shows (17). For example, the five-year survival of patients positive for GPC3 was considerably reduced compared to that of GPC-3-negative patients (54.5 *vs* 87.7%, P = 0.031), with this association between GPC3 level and HCC prognosis demonstrated in many studies (29). The early identification of GPC3-positivity may also predict tumor recurrence after resection, and GPC3 is recognized as an independent prognostic factor for disease-free survival (DFS) (30). A raised serum level of the GPC3 N-terminal subunit antigen (sGPC3N) has also been shown to be independently related to both OS (p < 0.05) and DFS (p < 0.01) (31). Furthermore, both GPC3 and osteopontin (OPN) overexpression are linked to reduced DFS in HBV-positive small HCC, with elevated levels of both molecules indicative of adverse outcomes after curative resection (32). For HCV-positive patients after surgical resection, GPC3 is a prognostic indicator for reduced DFS (33). Consistently, raised levels of GPC3 mRNA have been linked to the development of HCC after liver transplantation (34). Furthermore, a viable GPC3-based immunomagnetic fluorescent system (C6/MMSN-GPC3) has been developed to identify circulating tumor cells (CTCs) in HCC patients’ blood, further contributing to the early diagnosis and determination of prognosis (35).

## 3 GPC3-Associated Signaling Pathways in HCC

### 3.1 Wnt Signaling Pathway

Wnt signaling plays a major part in HCC pathology and is implicated in cell survival, proliferation, migration, and invasion. The first step in the pathway is the binding of Wnt to the membrane receptor Frizzled (FZD). Wnt signaling involves both canonical and non-canonical pathways, with the former involving the β-catenin protein (36, 37). β-catenin influences the expression of numerous genes, some of which are associated with cell proliferation and survival (38). GPC3 activates the canonical pathway, thereby stimulating HCC progression (39, 40). The human monoclonal anti-GPC3 antibody, HS20, binds the GPC3 HS moiety and has been shown to block the interaction between GPC3 and Wnt3a (41). GPC3 also interacts with FZD through the HS chain, suggesting that GPC3 may form a signaling complex with both FZD and Wnt (42). The N-leaf cysteine-rich domain (CRD) of GPC3 has a Wnt-binding groove, and the mutation of the notch reduces binding, thereby reducing Wnt activation, and inhibiting the growth of mouse liver cancer (43).

### 3.2 Other Signaling Pathways

**Figure 3:**
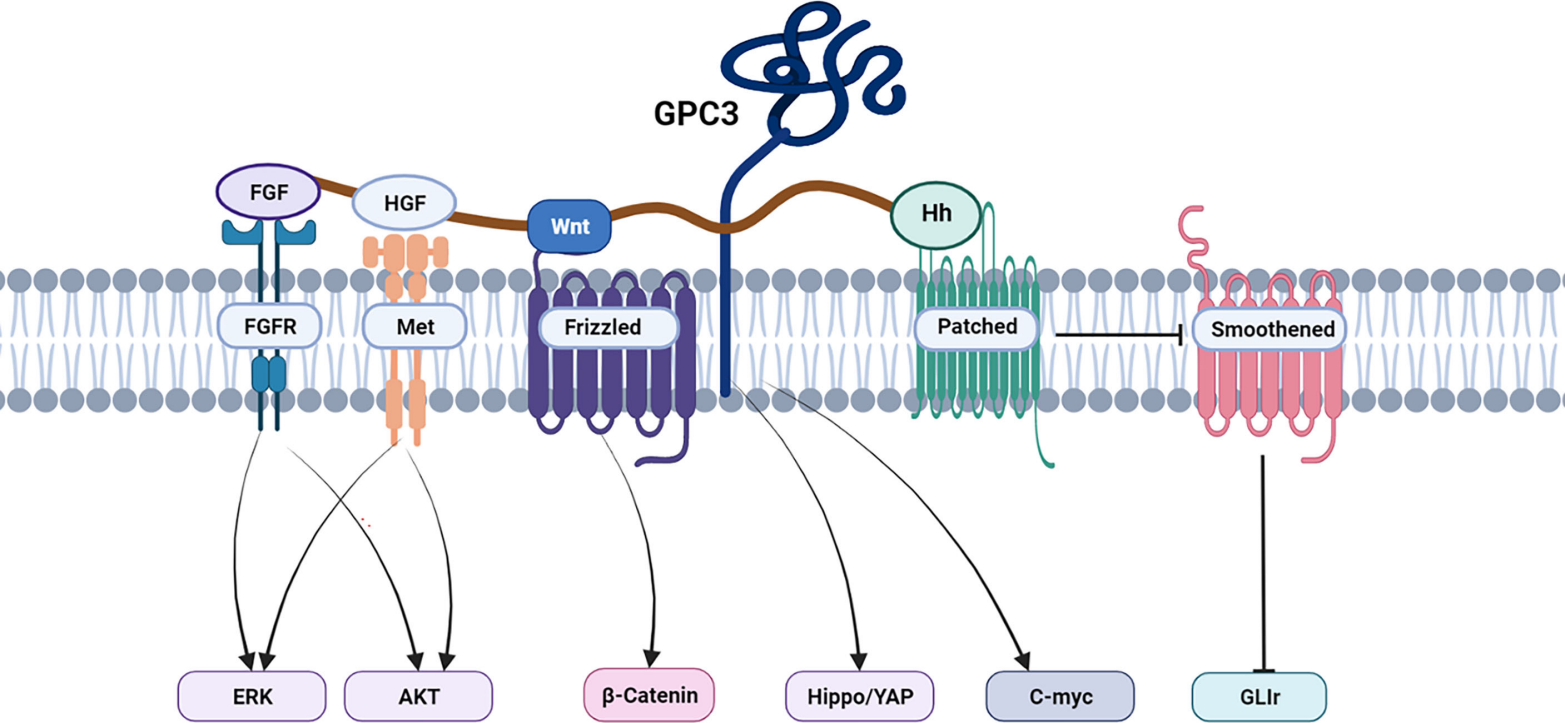
GPC3 associated signaling pathways in HCC.

The Hippo signaling pathway is responsible for reducing cell contacts and limiting both organ size and tumorigenesis (44). The Hippo pathway is frequently activated in HCC, with activation of the Yes-associated protein (YAP) (45, 46). GPC3 knockout inhibits YAP expression at both the mRNA and protein levels and induces the apoptosis of Huh7 cells (47, 48). In addition, the abnormal persistence of hedgehog signaling has been directly related to HCC (49–51), and GPC3 appears to be a negative regulator of hedgehog signaling (52–54). Transcription factors zinc-fingers and homeoboxes 2 (ZHX2) (55) and C-myc (56) are involved in the oncogenic activation of GPC3 in HCC to modulate HCC cell growth, proliferation, and differentiation. Sulfatase 2 may up-regulate GPC3 expression, promote fibroblast growth factor (FGF) signal transduction, and reduce the survival rate of HCC patients. A human monoclonal antibody against the GPC3 HS chain inhibited HGF/c-Met pathway-mediated migration and motility in hepatoma cells (41, 57–59). Furthermore, GPC3 could promote the progression and metastatic spread of HCC by influencing the functioning of tumor-associated macrophages (TAM) through macrophage recruitment (60). In **Figure 3**, we summarize the signal pathways related to GPC3.

## 4 GPC3 Targeted Therapy for HCC

**Figure 4:**
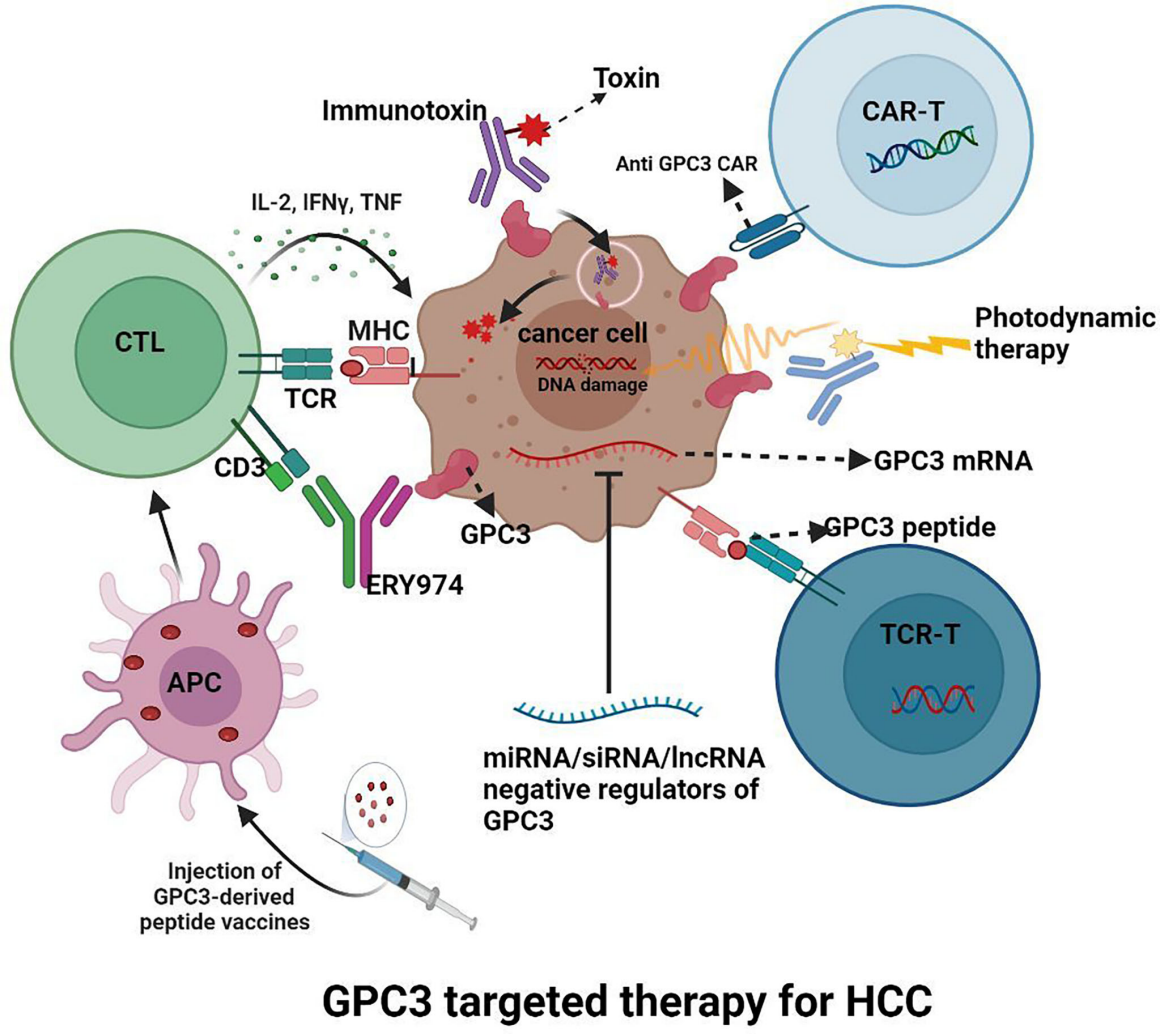
GPC3 targeted therapy for HCC.

Since GPC3 is overexpressed in HCC, as **Figure 4** shows, various inhibitors targeting GPC3 are under investigation.

### 4.1 GPC3-Targeted Antibodies

#### 4.1.1 Monoclonal Antibodies

GC33 is a recombinant, humanized, high-affinity monoclonal antibody against the GPC3 C-terminus. In preclinical assessments, GC33 was found to promote antibody-dependent cellular cytotoxicity (ADCC) in an antigen-dependent manner (61). The antibody also reduced tumor growth in xenograft models, with the growth reduction correlated approximately to the cell surface antigen level (61). In clinical application, Zhu, et al. enrolled 20 patients in a dose-escalation study, showing no dose-limiting toxicities (DLT) as the maximum tolerated dose fell beyond even the highest dose planned. This suggested the potential clinical efficacy and benefit of GC33 and warrants further evaluation. The minimum serum concentrations of the antibody were above the target concentrations at doses above 5 mg/kg and there was a significant reduction in the median time to progression (TTP) between the high-GPC3 group and the low-GPC3 group (26.0 weeks vs 7.1 weeks; P = 0.033) (62). Ikeda, et al. enrolled seven patients in a similar study in Japan to evaluate the safety and tolerability of GC33. They observed that GC33 was well-tolerated overall, with no DLTs and with the maximum tolerated dose (MTD) not reached. These findings are consistent with those of the First-in-Human although the small sample size did not allow a clear correlation between GPC3 expression and antitumor action (63). Recently, a double-blind, phase II trial of GC33 in 185 patients with chemotherapy-unresponsive HCC showed that, although Codrituzumab therapy itself was ineffective, when increasing Codrituzumab exposure, the levels of GPC3 and CD16 in circulating immune cells could predict the efficacy of the drug, suggesting that precision Codrituzumab therapy with this perspective may have potential for treating HCC (64).

Another monoclonal antibody, 32A9, specifically targeting the middle region of GPC3, reduced the growth of HCC tumors in mice. This study then investigated two 32A9-based immunotherapeutic strategies involving an immunotoxin and CAR-T cells. It was found that the antibody-immunotoxin complex was specifically cytotoxic to GPC3-positive tumor cells, while the 32A9-CAR-T cells destroyed the tumor cells *in vitro* and promoted regression of HCC xenograft tumors *in vivo* (65). Feng et al. described an antibody, HN3, that recognized the full GPC3 molecule with high affinity. The antibody promoted cell cycle arrest in G1, inhibiting the growth of GPC3-expressing cells and reducing the growth of xenografts in mice (66). Another human anti-GPC3 monoclonal antibody, HS20, that recognizes the HS moiety on the molecule, was shown to block Wnt signaling and inhibit tumor growth. This antibody also showed no toxicity in mice (41). Thus, although GPC3 is a well-characterized HCC-associated antigen, anti-GPC3 therapeutic strategies have had limited clinical success.

#### 4.1.2 Bispecific Antibodies

Given the low clinical response rate of monoclonal antibodies targeting GPC3, bispecific antibodies have been investigated. One such bispecific antibody, ERY974, a humanized IgG–structured T cell–redirecting antibody (TRAB) with a common light chain, could bind to both GPC3 and CD3, promoting cytotoxicity through the action of T cell effectors. ERY974 also showed significant non-immunogenic antitumor effects in tumors that were unresponsive to treatment with immune checkpoint (such as PD-1 and CTLA-4) inhibitors. Further investigation showed that ERY974 induced a high degree of inflammation in the tumor microenvironment, with toxicology studies in cynomolgus monkeys showing raised levels of cytokines in the short-term (67). A further report demonstrated a significant improvement in antitumor action in xenograft models using a combination of ERY974 and chemotherapy (68). A phase I clinical trial of this antibody is ongoing (NCT02748837). GPC3/CD47, a bispecific antibody targeting GPC3 and CD47, was effective in preventing tumor growth through recognition of both antigens. This antibody has a long serum half-life with no adverse systemic effects compared to an anti-CD47 antibody alone. The antibody was more effective than treatment with a single anti-CD47 antibody or a combination of individual anti-CD47 and anti-GPC3 antibodies in a mouse xenograft model (69). Taken together, these results suggest that anti-GPC3 bispecific antibodies might be potential therapeutic treatments for HCC in the future.

### 4.2 GPC3-Derived Peptide/DNA Vaccines

In addition to antibodies targeting GPC3, the application of GPC3-derived peptide/DNA vaccines is another potentially attractive option for treating HCC. Nakatsura, et al. showed that both HLA-A24(A*2402)-restricted and H-2Kd-restricted GPC3298–306 peptide (EYILSLEEL) peptides, as well as the HLA-A2(A*0201)-restricted GPC3_144–152_ peptide (FVGEFFTDV), can induce GPC3-reactive cytotoxic T lymphocytes (CTLs) (70, 71). These peptides were subsequently tested as vaccines in preclinical trials using mouse models, and schedules for clinical trials were set up, testing the GPC3298–306 and GPC3144–152 peptides in a Phase I clinical trial. In the trial, one patient showed a partial response (PR), while 4 out of 19 patients with stable disease (SD) showed tumor regression or necrosis beyond the PR criteria. After two months of ongoing treatment, the disease control rate (PR+SD) was 60.6% (72). A pilot study (UMINCTR: 000005093) confirmed lymphocyte tumor infiltration by after vaccination with the GPC3 peptides. A Phase II, open-label, single-arm trial (UMIN-CTR: 000002614) enrolled 40 HCC patients who had received either surgery or radiofrequency ablation. In the year following curative treatment, 10 vaccinations were administered, resulting in a significantly lower recurrence rate in patients who had received surgery/radiofrequency ablation with vaccines than in patients who had been treated with surgery only (73). Intravenous administration of GPC3-coupled lymphocytes (LC/GPC3^+^) resulted in the production of both anti-GPC3 antibodies and CTLS, reducing HCC growth and lysing HCC cells in culture (74). Apart from these peptide vaccines, GPC3 DNA vaccines could elicit CTL responses against HCC cell lines, inhibit homogenous tumor growth, and increase the survival rates of xenograft-bearing mice (75). However, despite the potential attraction of a peptide vaccine, the antitumor effects are too weak for treating advanced HCC. Intratumoral peptide injection or combining the peptide vaccine with an anti-PD-1 blocking antibody could feasibly enhance the antitumor effects.

### 4.3 Immunotoxins

GPC3-targeted human nanobody (HN3) immunotoxins have been reported to have potent antitumor effects through the blocking of protein synthesis and downregulation of the Wnt signaling pathway. For example, it was found that intravenously administering the immunotoxin HN3-PE38 either individually or in combination with chemotherapeutic drugs promoted regression of Hep3B and HepG2 tumor xenografts in mice. These results indicate the potential of GPC3 use in immunotoxin-based treatment. However, a drawback was that the side effects and potential toxicity of the immunotoxin, which could thus only be used at low doses (< 0.8 mg kg-^1^) (76). In addition, another team of researchers constructed two mPE24-based immunotoxins (HN3-mPE24 and HN3HN3-mPE24). HN3-mPE24 had both high-affinity antigen-binding and strong anti-tumor effects in HCC cells, with minimal side effects in mice even at high doses, and resulted in effective tumor regression and improved survival rates. However, immunogenic effects and the relatively short half-lives of immunotoxins may limit their clinical application (77). To overcome this shortcoming, another research team engineered HN3-ABD-T20 and HN3-ALB1-T20 by adding an albumin-binding domain (ABD) to prolong their half-life. This resulted in effective tumor regression at one-tenth of the dose required for HN3-T20. This increased potency was ascribed to the observed 45-fold prolongation of HN3-ABD-T20’s serum retention time. Pharmacokinetic studies in mice showed that HN3-ABD-T20 had a half-life of about 5.5 hours compared to only 7 minutes for HN3-T20. HN3-ABD-T20 thus represents the best option for clinical translation because of its long serum retention, high cytotoxicity, and reduced antigenicity (78). Although further investigations, including clinical trials, are required, these findings suggest that GPC3-targeted immunotoxins have promising potential for treating HCC.

### 4.4 GPC3 CAR-T/NK Cells

In recent years, CAR-T cell therapy has proved effective for treating several cancers, especially hematological malignancies (79, 80). To date, there have been several clinical trials exploring the use of GPC3 CAR-T in HCC (**Table 1**). GPC3-targeted CAR-T cells are able to destroy GPC3^+^ HCC cells *in vitro* and GPC3^+^ HCC tumor xenografts in mice. Combinations of sorafenib and GPC3-CAR T cells have also proved effective (81). Compared with GPC3-CAR-T cells, the combination of GPC3 and epidermal growth factor receptor (EGFR)-dual-targeting CAR-T cells is more effective in reducing HCC growth (82). To further increase the specificity and decrease the off-target risk, IL-12-armored GPC-3-redirected CAR-T cells were designed which showed greatly improved antitumor effects in mouse models (83). An IL-4/21 inverted cytokine receptor also improved CAR-T cell potency in an immunosuppressive tumor microenvironment (84). GPC3-specific CAR-T cells co-expressing IL-15 and IL-21 (85) or IL-7 and PH-20 (86) were found to be effective against HCC. Interestingly, disruption of PD-1 gene expression in GPC3 CAR-T cells by the CRISPR/Cas9 gene-editing system increased the *in vivo* activity of CAR-T cells against HCC, improving their infiltration levels in mouse models (87). Co-stimulation of DNAX-activating protein 10 was shown to increase the anti-tumor action of CAR-T cells (88). Interestingly, shed GPC3 competed with cell-surface GPC3 CAR-T cell binding, inhibiting the effects of the cells in HCC (89).

**Table 1:** Clinical trials of GPC3-CAR-T for treating liver cancer.

Interventions	Study Title	Trial No.	status	Phase	Locations
**Monotherapy**					
GPC3 CAR-T cells	GPC3 CAR-T cells in patients with refractory HCC	NCT03146234	Completed	Not Applicable	Shanghai, China
	CAR-T Cells Targeting GPC3	NCT03884751	recruiting	1	Zhejiang, China
	4th generation CAR-T cells targeting GPC3	NCT03980288	recruiting	1	Zhejiang, China
	GPC3 CAR-T Cells for the Hepatocellular Carcinoma	NCT04506983	a Not yet recruiting	1	Beijing, China
	A Study of GPC3-targeted T Cells by Intratumor Injection for Advanced HCC (GPC3-CART)	NCT03130712	Unknown	1/2	Beijing, China
	A Study of GPC3 Redirected Autologous T Cells for Advanced HCC	NCT02715362	Unknown	1/2	Shanghai, China
	GPC3-CAR-T Cells for Immunotherapy of Cancer With GPC3 Expression	NCT03198546	recruiting	1	Guangdong, China
	A Study of Chimeric Antigen Receptor T Cells Combined With Interventional Therapy in Advanced Liver Malignancy	NCT02959151	Unknown	1/2	Shanghai, China
	CAR-T Cell Immunotherapy for HCC Targeting GPC3	NCT02723942	Withdrawn	1/2	Guangdong, China
	GPC3-targeted CAR-T Cell for Treating GPC3 Positive Advanced HCC	NCT04121273	recruiting	1	Jiangsu, China
anti-GPC3 CAR-T	Anti-GPC3 CAR T for Treating Patients With Advanced HCC	NCT02395250	Completed	1	Shanghai, China
**Combined chemotherapy**					
GAP T cells, Cytoxan, Fludara	GPC3-specific Chimeric Antigen Receptor Expressed in T Cells for Patients With Pediatric Solid Tumors (GAP)	NCT02932956	Recruiting	1	Texas, United States
AGAR T cells, Cytoxan, Fludara	Interleukin-15 Armored GPC3-specific Chimeric Antigen Receptor Expressed in T Cells for Pediatric Solid Tumors	NCT04377932	Not yet recruiting	1	Texas, United States
CARE T cells, Cytoxan, Fludara	Interleukin-15 and -21 Armored Glypican-3-specific Chimeric Antigen Receptor Expressed in T Cells for Pediatric Solid Tumors	NCT04715191	Not yet recruiting	1	Texas, United States
TEGAR T cells, Cytoxan, Fludarabine	T Cells co- Expressing a Second Generation GPC3-specific Chimeric Antigen Receptor With Cytokines Interleukin-21 and 15 as Immunotherapy for Patients With Liver Cancer (TEGAR)	NCT04093648	Withdrawn	1	Unknown
GLYCAR T cells, Cytoxan, Fludarabine	GPC3-specific Chimeric Antigen Receptor Expressing T Cells for Hepatocellular Carcinoma (GLYCAR)	NCT02905188	Recruiting	1	Texas, United States
Retroviral vector-transduced autologous T cells to express anti-GPC3 CARs, Fludarabine, Cyclophosphamide	Anti-GPC3 CAR-T for Treating GPC3-positive Advanced Hepatocellular Carcinoma (HCC)	NCT03084380	Unknown	1/2	Chongqing, China
**Combined with other immunotherapy**					
CAR-CD19 T cell, CAR-BCMA T cell, CAR-GPC3 T cell, (and 3 more…)	Clinical Study of Redirected Autologous T Cells With a Chimeric Antigen Receptor in Patients With Malignant Tumors	NCT03302403	Active, not recruiting	Not Applicable	Zhejiang, China

(U.S. National Library of Medicine | U.S. National Institutes of Health | U.S. Department of Health & Human Services)(Updated to 2021).

There are, however, side effects in the use of CAR-T cells, including tumor lysis syndrome, cytokine release syndrome, and on-target, off-tumor effects. These side-effects, rather than the neoplasm itself, may even be fatal. NK-92 cells have been developed to incorporate improved efficacy with minimal toxicity. The safety and cytotoxic specificity of genetically modified NK-92 cells have been attested to in preclinical trials, suggesting that these cells may be ideal carriers for CAR (90). The anti-tumor efficacy of NK-92/9.28.z cells has been confirmed in many HCC xenografts with different GPC3 levels (91). The combination of CAR-T and GPC3-targeted treatments appear to be highly promising, especially if combined with ICBs.

### 4.5 Gene Therapy

The use of gene therapy targeting GPC3 has also been investigated. For example, sulfatase 2 (SULF2) knockdown decreased HCC cell proliferation and migration as well as xenograft growth (58). MicroRNAs (miRNAs) targeting GPC3 have been described, with low levels of miR-1271 related to GPC3 overexpression in HCC, with the miRNA reducing HCC cell growth in a GPC-3-dependent manner and inducing cell death (92). However, although the siRNA technology is effective for the specific silencing of individual genes, it is difficult to apply to a clinical setting as it requires effective delivery with high specificity and minimal toxicity. A GPC3-targeted siRNA nanovector (NP-siRNA-GPC3 antibody for HCC treatment) showed obvious antitumor efficacy *in vitro* with minimal toxicity and significantly inhibited orthotopic HCC xenografts (93). It is known that long non-coding RNAs (lncRNAs) play significant roles in cancer, including HCC. Knockdown of the HOXA cluster antisense RNA2 (HOXA-AS2) lncRNA reduced GPC3 expression and blocked HCC cell proliferation by G1 arrest, as well as promoting apoptosis and inhibiting HCC cell migration and invasion *in vitro* (94).

### 4.6 Combination of Anti-GPC3 and ICIs

Combining anti-GPC3 antibodies and immune checkpoint inhibitors (ICIs) may be a promising strategy for GPC3-associated cancers. For example, treatment with the GC33 antibody increased the infiltration of PD-L1 positive immune cells (such as macrophages and multinucleated giant cells), and mGC33 combined with an anti-mPD-L1 monoclonal antibody was more effective against tumors than the antibody alone in xenograft HCC models (95). A Phase I clinical trial of the anti-GPC3 monoclonal antibody Codrituzumab combined with atezozumab showed that the agents were well-tolerated and effective in reducing tumor growth in patients with advanced HCC. Among 18 evaluable patients, 1 case was diagnosed as PR, and 10 were SD (including 1 case of unconfirmed PR), of which 6 cases had SD more than 6 months before progression. No DLT was observed (96). Thus, GPC3-CAR-T in combination with anti-PD-1 has increased antitumor efficacy and may have potential for the treatment of HCC patients (97, 98).

### 4.7 Other Therapies

The direction of T cells to tumors is important in cancer therapy. For example, T cells combined with GPC3-specific antibodies are able to destroy GPC3-expressing HCC xenograft tumors in mice (99). Photodynamic therapy (PDT) is a novel method for treating tumors; this relies on the production of reactive oxygen species that induce tumor cell death. This is linked to both vascular shutdown and enhancement of immune activity, but its applications have been limited by the poor tissue penetration of visible light. The use of the near-infrared (NIR) photosensitizer may solve these limitations (100). For example, UCNPs@mSiO2-Ce6-GPC3 nanoparticles are biocompatible, have low toxicity, and produce good cell imaging and antitumor results (101). A novel multi-functional nanostructure, galactose (GAL)- golden nanorods (GNR)-siRNA of GPC3(siGPC3) was found to produce both silencing of the GPC3 gene and photothermal action, and may be useful as a synergistic treatment for cancer (102). A study on a GPC3-targeting peptide (named G12)-modified liposome (GSI-Lip) co-loaded with sorafenib (SF) and IR780 iodide (IR780) showed promising sensitivity and specificity in detecting HCC together with synergistic effects on chemo-photothermal theranostics (103). Thus, the combination of chemotherapeutic drugs and siRNA may have potential in improving anticancer effects using synergistic interactions. SF-PL/siGPC3 with selected sizes and zeta potentials, delivered by PEI-modified liposomes, was shown to accumulate at the tumor site and to enter HCC cells, resulting in suppression of both GPC3 and the pro-proliferation gene cyclin D1 expression a. Intravenous injection of SF-PL/siGPC3 into HepG2-bearing nude mice both blocked tumor growth and prolonged survival (104). GPC3 is involved in the progression of HCC, including stimulation of Wnt signaling, Hedgehog signaling. MiR-542-3p (105) and miR-485-5p (106) block the Wnt signaling pathway, while GANT61 (107) and bufalin (108) affect the Hedgehog signaling pathway to inhibit HCC.

### 4.8 Toxicities for Targeting GPC3

While exhibiting great efficacy, toxicities for targeting GPC3 must be attention. In GPC3 antibody therapy, GC33 was well tolerated in HCC, the most common adverse events (AEs) were the decrease of lymphocyte count (77%) and NK cell count (77%), no grade 4 or 5 AEs were reported (63). When GC33 combined with anti-PD-L1 antibody, grade≥3 AEs were increased aspartate aminotransferase and decreased lymphocyte count (96). Although the phase I clinical data of ERY974 have not been published, in animal trials, the most prominent AEs is cytokine release syndrome (CRS), an acute inflammatory syndrome resulted from the activation of immune cells and release of pro-inflammatory cytokines, however, cytokine release can be managed by corticosteroid premedication (67). In GPC3 vaccine therapy, there are reports of patients with tumor lysis syndrome after the second GPC3 peptide injection, which led to high fever, liver failure, and death (109). Thus, researchers need to optimize the balance between superior tumor-killing abilities and severe tumor lysis syndrome. In GPC3 CAR-T therapy, the commonest grade 3/4 adverse event was hematotoxicity, mainly due to transient lymphocyte count reduction resulted from lymphatic depletion (110, 111). Moreover, cytokine release syndrome (CRS), an acute inflammatory syndrome resulted from the activation of immune cells and release of pro-inflammatory cytokines, should be taken seriously. In a phase I clinical trial of GPC3 CAR-T for HCC, CRS occurred in 9/13 patients, including 1 case of grade 5 CRS (died on day 19) (110). In another study, CRS occurred in all patients, with a 50% incidence of grade≥3 CRS (3/6) (111). In addition, neurotoxicity is related to CRS, cytokines are elevated not only in blood, but also in cerebrospinal fluid, and its clinical symptoms mainly include headache and disturbance of consciousness (112). Fortunately, administrate high-dose corticosteroids or IL-6 receptor antagonist drug tocilizumab was able to alleviate CRS (113). In patients with high tumor load, there is a more severe CRS (114).The use of CAR-T either in the early stage of disease course or after reducing tumor burden may significantly reduce the risk of severe CRS. Despite the low expression of GPC3 in normal adult tissues (115), “on-target off-tumor” may lead to disastrous side effects. GPC3 is expressed in placenta and endometrium (116, 117), suggesting that female patients, especially pregnant patients, may have a high risk of “on-target off-tumor”. Furthermore, a small amount of GPC3 was expressed in normal renal tubular and testicular germ cells (115), so renal function should be monitored during targeted GPC3 treatment, and reproductive protection should also be paid attention to in infertile men. At present, assembling suicide genes, synthetic notch receptors, on-switch CAR, bispecific CAR-T cells can help prevent healthy cells from CAR-T attacking (118). At present, no obvious toxicity has been reported in GPC3 related gene therapy, immunotoxin and photodynamic therapy (78, 93, 101).

## 5 Conclusion

Hepatocellular carcinoma has an extremely poor survival rate. To improve both the outcome and quality of life of these patients, it is necessary to discover and develop new means of treating the disease. GPC3 is specifically associated with liver cancer and, although it is useful in HCC diagnosis, an individual marker is not able to meet the needs of clinical therapeutic application. While using a panel of multiple markers greatly improves the rate of early cancer detection, this only strengthens the suspected diagnosis of HCC, so further exploration into increasing the sensitivity and specificity of these markers is required.

GPC3 has exceptional cancer specificity and is currently being investigated as a global target for cancer-targeted therapies and immunotherapies. A series of antibodies against HCC is currently in clinical and preclinical trials. However, single anti-GPC3 antibody therapy does not kill liver cancer altogether, which may need to achieve high target saturation in tumor cells to induce any beneficial effect. Bispecific antibodies recognize different epitopes on the antigen simultaneously, overcoming the shortcomings of traditional monoclonal antibodies and showing excellent results in animal experiments, but these results require verification in clinical trials; nevertheless, the promising results suggest the potential of developing combined immunotherapies by optimizing antibody structures and raising antibodies against multiple targets. Second-generation GPC3-based immunotherapies, such as CAR-T and TCR engineering T cell therapy, have attracted worldwide attention. CAR-T can effectively kill tumor cells with low expression of cell surface antigens, which will expand substantially in the body during treatment of patients. However, CAR-T cells only show moderate anti-tumor activity in patients with solid tumors, including liver cancer, partly because of their specific immune microenvironment, containing the vascular-stromal barrier reduces the expansion, persistence and penetration of CAR-T; immune checkpoints and immunosuppressive cells allow HCC to undergo immune escape (119). The CAR co-expressing IL-15 and IL-21 showed improved activity. In addition, the toxicity caused by CAR-T has limited the application. Therefore, optimization of the CAR structure to enhance the *in vivo* peak expansion and safe half-life of CAR-T warrants further investigation. It is also possible that the surviving cells may cease to express GPC3 during the treatment, resulting in drug resistance. GPC3-negative tumors may also grow and develop drug resistance under such therapeutic pressure. Therefore, the exploration of novel targets and combination therapies are future goals for HCC research.

## Author Contributions

XZ and XL gathered information and designed the review. YL drew the pictures. ML and GW critically revised the manuscript. All authors contributed to the article and approved the submitted version.

## Funding

This work was supported by the National Natural Science Foundation of China (31971390), Sichuan Science and Technology Program (2021YFH0142), and 1.3.5 Project for Disciplines of Excellence, West China Hospital, Sichuan University (Grant No. ZYJC21043).

## Conflict of Interest

The authors declare that the research was conducted in the absence of any commercial or financial relationships that could be construed as a potential conflict of interest.

## Publisher’s Note

All claims expressed in this article are solely those of the authors and do not necessarily represent those of their affiliated organizations, or those of the publisher, the editors and the reviewers. Any product that may be evaluated in this article, or claim that may be made by its manufacturer, is not guaranteed or endorsed by the publisher.
